# Carnosine inhibits glioblastoma growth independent from PI3K/Akt/mTOR signaling

**DOI:** 10.1371/journal.pone.0218972

**Published:** 2019-06-27

**Authors:** Henry Oppermann, Helene Faust, Ulrike Yamanishi, Jürgen Meixensberger, Frank Gaunitz

**Affiliations:** Klinik und Poliklinik für Neurochirurgie, Universitätsklinikum Leipzig AöR, Leipzig, Germany; Duke University School of Medicine, UNITED STATES

## Abstract

Glioblastoma is a high-grade glioma with poor prognosis even after surgery and standard therapy. Here, we asked whether carnosine (β-alanyl-L-histidine), a naturally occurring dipeptide, exert its anti-neoplastic effect on glioblastoma cells via PI3K/Akt/mTOR signaling. Therefore, glioblastoma cells from the lines U87 and T98G were exposed to carnosine, to the mTOR inhibitor rapamycin and to the PI3K inhibitor Ly-294,002. Pyruvate dehydrogenase kinase (PDK4) expression, known to be a target of PI3K/Akt/mTOR, and which is also affected by carnosine, was analyzed by RT-qPCR, and reporter gene assays with the human PDK4 promoter were performed. Cell viability was assessed by cell-based assays and mTOR and Akt phosphorylation by Western blotting. Rapamycin and Ly-294,002 increased PDK4 mRNA expression in both cell lines but significance was only reached in U87. Carnosine significantly increased expression in both lines. A significant combinatorial effect of carnosine was only detected in U87 when the dipeptide was combined with Ly-294,002. Reporter gene assays revealed no specific effect of carnosine on the human PDK4 promoter, whereas both inhibitors increased reporter gene expression. Rapamycin reduced phosphorylation of mTOR, and Ly-294,002 that of Akt. A significant reduction of Akt phosphorylation was observed in the presence of carnosine in U87 but not in T98G, and carnosine had no effect on mTOR phosphorylation. Cell viability as determined by ATP in cell lysates was reduced only in the presence of carnosine. We conclude that carnosine’s anti-neoplastic effect is independent from PI3K/Akt/mTOR signaling. As the dipeptide reduced viability in tumor cells that do not respond to PI3K or mTOR inhibitors, it appears to be worth to further investigate the mechanisms by which carnosine exerts its anti-tumor effect and to consider it for therapy, especially as it is a naturally occurring compound that has already been used for the treatment of other diseases without indication of side-effects.

## Introduction

The most common and aggressive primary tumor of the brain is designated Glioblastoma (GBM). It is classified by the WHO (World Health Organization) as grade IV glioma. In the United States it accounts for 46.6 percent of all malignant tumors of the central nervous system (CNS) and for 55.4 percent of gliomas. Its incidence is 3.21 per 100.000 population which accounts for 13,010 cases projected in 2018 and 13,310 cases projected in 2019 [1]. Current standard of medical treatment after maximal safe resection is radiotherapy and adjuvant chemotherapy with the alkylating agent temozolomide. Unfortunately, in patients with newly diagnosed GBM the median survival under this treatment is only 12 to 15 month [2,3] and there is urgent need for new strategies for treatment including targeted and immunotherapy strategies (for recent reviews see [4,5]). Among the intracellular pathways, which are intensively investigated as potential targets for treatment strategies, is the PI3K/AKT/mTOR pathway (Phosphoinositide 3-kinase/Ak strain transforming/mechanistic target of rapamycin pathway) (for review see [6]). The more than 50 PI3K inhibitors that have been designed for cancer treatment are classified into pan-PI3K, isoform selective or dual PI3K/mTOR inhibitors (for a recent review see [7]). The drugs MK-2206 and perifosine (KRX-0401) are used as inhibitors of Akt and there is a number of inhibitors of mTOR currently investigated, including temsirolimus, sirolimus (rapamycin), everolimus and ridaforolimus (for review see: [5]).

In recent years our group investigated whether the dipeptide carnosine (β-alanyl-L-histidine) is a candidate for glioblastoma therapy. Carnosine has originally been isolated from Liebig’s meat extract almost 120 years ago by Gulewitsch and Amiradzibi [8]. The dipeptide is highly abundant in skeletal muscle with around 20±4.7 mmol per kg dry weight [9], and since its discovery several physiological properties have been ascribed to it, such as pH-buffering, scavenging of reactive oxygen species and heavy metal ions, protection from lipid peroxidation and ischemic brain damage (for a comprehensive review see [10]). Furthermore, an anti-neoplastic effect of carnosine has been demonstrated in vitro and in vivo in a number of cancer models, such as human colon carcinoma [11], gastric carcinoma [12], cervix carcinoma [13], and GBM [14,15]. Interestingly, the dipeptides effect is not limited to proliferation and cell cycle control, but it is also able to decrease the migration of glioblastoma cells [16]. In fact, migration and invasive behavior are hallmarks of glioblastoma, leading to recurrence of tumors only a few months after surgical removal of the primary tumor mass.

Unfortunately, not much is known about the molecular targets of carnosine in cancer cells. Among the mechanisms discussed are an influence on ATP production from glucose [17], and there are observations that depending on the model investigated, hypoxia inducible factor 1 (HIF1) [18], the signal transducer and activator of transcription 3 (STAT3) [19], the mitogen-activated protein kinase (MAPK) [20], and the Kirsten rat sarcoma viral oncogene (KRAS) [21] might be influenced. In addition, there have been hints that the Akt/mTOR/Ribosomal protein S6 kinase beta-1 pathway is involved in carnosine’s anti-neoplastic effect [22]. At this point, it is noteworthy, that other observations also point towards the possibility that carnosine may be a mimic of rapamycin, which is an inhibitor of mTORC1 (mTOR complex 1) [23]. In addition, carnosine’s anti-neoplastic effect in glioblastoma cells was shown to be accompanied by increased expression of pyruvate dehydrogenase kinase 4 (PDK4) [24]. As PDK4 expression is regulated by transcription factors, such as FOXO1a and FOXO3a (Forkhead box protein O1a and O3a) [25], which are downstream effectors of PI3K/AKT/mTOR signaling, we hypothesized that carnosine’s anti-neoplastic effect may be mediated by an influence on this pathway. Therefore, we compared the effect of carnosine on glioblastoma cell viability, PDK4 expression and signaling molecule phosphorylation with the PI3K inhibitor Ly294,002 and the mTOR inhibitor rapamycin in two cell lines derived from glioblastoma.

## Materials and methods

### Reagents

If not stated otherwise, all chemicals were purchased from Sigma Aldrich (Taufkirchen, Germany) including the PI3K inhibitor Ly-294,002, and the carnosine employed in this study (Cat.-Nr.: C9625/ Lot: BCBK4678V). The mTOR inhibitor rapamycin was purchased from Santa Cruz Biotechnology (Heidelberg, Germany).

### Cell culture

U87 (ATCC HTB-14) and T98G (ATCC CRL-1690) cells were obtained from the American Type Culture Collection (ATCC, Manassas, USA) and genotyped (Genolytic GmbH, Leipzig, Germany) to confirm their identity prior to the experiments. Cells were propagated in 250 ml culture flasks (Sarstedt AG & Co., Nümbrecht, Germany) using 10 ml of standard culture medium (SCM) consisting of DMEM (Dulbecco’s Modified Eagle Medium) with 4.5 g/l glucose, and without pyruvate (Life Technologies, Darmstadt, Germany), supplemented with 10% fetal bovine serum (FBS superior, Biochrom, Berlin, Germany), 2 mM GlutaMAX (Life Technologies) and Penicillin-Streptomycin (Life Technologies) at 37°C and 5% CO_2_ in humidified air in an incubator.

### Reporter gene construction

The human PDK4 promoter (-3968/+319 bp relative to the transcription start site) was obtained by PCR (polymerase chain reaction) employing the GoTaq Long PCR Master Mix (Promega, Mannheim, Germany) using genomic DNA extracted from T98G cells (QIAamp DNA mini Kit, Qiagen) as template using the following primers: *hPDK4_(-)3968*: 5’-CAT GGC GGG ATC CTT TCT TAT GGG CTG C-3’; (forward) and *hPDK4_(+)319*: 5’-CGC CTC CAT GGT GAC GCC CAC CC-3’ (reverse). *hPDK4_(-)3968* was designed containing a *Bam*HI recognition site and *hPDK4_(+)319* containing an *Nco*I recognition site (both underlined in the primer sequences presented). These recognition sites allowed subcloning of the amplification product into the reporter gene *pT81_GauIII* (containing the secreted luciferase from *Gaussia princeps* [26]) after restriction digestion of both the PCR product and the vector. In this way the reporter gene *hPDK4_GauIII* was obtained in which a ~4000 bp 5’-region (-3986/+319) from the PDK4 gene controls the luciferase from *Gaussia princeps* with the endogenous PDK4 start codon matching the start codon of the luciferase gene.

### Reverse transcription–quantitative real time polymerase chain reaction experiments

For the quantification of mRNA, RT-qPCR (reverse transcription–quantitative real time polymerase chain reaction) experiments were carried out as described [24]. Briefly, 10^6^ cells were seeded into 100-mm cell culture dishes (TPP, Trasadingen, Switzerland) with 10 ml of medium. After 24 hours of incubation, cells received fresh medium containing the compounds to be tested. Cells were harvested 24 hours later and RNA was isolated using a miRNeasy mini kit (Qiagen, Hilden, Germany) according to manufacturer’s instructions. The RNA was stored at -80°C until further use. 500 ng of RNA were used for reverse transcription employing the ImProm-II Reverse Transcription System (Promega, Mannheim, Germany) according to manufacturer’s instructions using random primer sets. DNA amplification was performed on a Rotor-Gene 3000 system (Qiagen) employing SYBR Green (Maxima SYBR Green/ROX qPCR Master Mix, Thermo scientific). Copy numbers of individual mRNAs were determined using linearized plasmid DNA (described in [24]) containing the corresponding target sequence. Data analysis was performed using the Rotor-Gene 6 software, and data was processed as described [24]. For reference, the transcript encoding the TATA-box binding protein (TBP) was used. Primer sequences: *PDK4_forward*: 5’-CTG TGA TGG ATA ATT CCC-3’; *PDK4_reverse*: 5’-GCC TTT AAG TAG ATG ATA GCA-3’; *TBP_forward*: 5’-TGA CCT AAA GAC CAT TGC AC-3’; *TBP_reverse*: 5’-GCT CTG ACT TTA GCA CCT GTT-3’.

### Cell viability assays

For the determination of ATP production the CellTiter-Glo Assay (CTG) and for the determination of dehydrogenase activity the CellTiter-Blue Assay (CTB) were employed (all from Promega, Mannheim, Germany) according to the instructions of the manufacturer, and as described previously [27]. All measurements of luminescence and fluorescence were performed using either a Mithras LB 940 Multimode Microplate reader (Berthold Technologies, Bad Wildbad, Germany) or a Spectra Max M5 reader (Molecular Devices, Biberach, Germany).

### Transfection experiments

Transient transfection was performed using TurboFect transfection reagent (Thermo scientific, Dreieich, Germany) according to manufacturer’s instructions. The ratio of DNA to TurboFect employed was 2 μg DNA per 4 μl transfection reagent. For transfection, cells were first seeded in 96-well plates at a density of 10,000 cells per well in 200 μl of medium. After 24 hours, old medium was removed and fresh medium containing the DNA/Polymer complexes was added (100 μl with an equivalent of 25 ng DNA). Three hours later, medium was exchanged and the cells received fresh medium with the supplements to be tested. *Gaussia* luciferase activity was determined from the supernatant 24 hours after the start of the transfection as described before [26]. Briefly, cell supernatant (5 μl) was transferred into a well of a white 96-well plate (Greiner bio-one, Frickenhausen, Germany). After an incubation of 20 minutes at room temperature, *Gaussia* luciferase activity was determined using a Mithras LB 940 Multimode reader (Berthold Technologies, Bad Wildbad, Germany) by injecting 50 μl of luciferase assay reagent (20 mM MOPS (3-(N-morpholino)propanesulfonic acid); 0.75 M KBr; 5 mM MgCl_2_; 5 mM CaCl_2_; 1 mM EDTA (ethylenediaminetetraacetic acid); 10 μM Coelenterazine; pH 7.8) to the cell supernatant, followed by a 1.6 second delay until luminescence was determined within a 0.5 s integral.

### Western Blot analysis

Western Blot experiments were performed with cells cultivated at a density of 10^6^ cells per dish in 100-mm cell culture dishes for 24 hours in the presence of the test compounds. Briefly, cells were washed twice with ice-cold washing buffer (137 mM NaCl; 5.4 mM HCl; 0.41 mM MgSO_4_, 0.49 mM MgCl_2_; 0.126 mM CaCl_2_; 0.33 mM Na_2_HPO_4_; 0.44 mM KH_2_PO_4_; 2 mM HEPES (4-(2-hydroxyethyl)-1-piperazineethanesulfonic acid); pH 7.4), and were then transferred to a 1.5 ml reaction vial using a cell scraper (TPP, Trasadingen, Switzerland), and 1 ml of ice-cold washing buffer. Cells were collected by centrifugation (500xg; 4 min; 4°C), and resuspended in 150 μl of ice-cold RIPA buffer (50 mmol/l Tris (tris(hydroxymethyl)aminomethane); 150 mmol/l NaCl; 1% Nonidet P40; 0.25% Natriumdesoxycholat; 0.1% SDS; pH 8.0) containing a combination of protease inhibitors (0.025 mg/ml Aprotinin; 0.025 mg/ml Leupeptin; 2.5 mmol/l Benzamidin; 0.01 mg/ml Pepstatin A; 2.5 mmol/l phenylmethylsulfonylfluorid), phosphatase inhibitors (PhosSTOP, Sigma) and 1 mmol/l DTT (Dithiothreitol). Cells were sonified using a Bioruptor system (Diagenode, Seraing, Belgium) at highest energy setting using a “30 s on/ 30 s off” protocol for 7.5 min. After sonification debris was removed by centrifugation (5500xg; 5 min; 4°C) and the supernatant containing the proteins was transferred to fresh reaction vials. Proteins were either immediately used for Western blotting or frozen at -80°C for long term storage.

SDS-PAGE (sodium dodecyl sulfate–polyacrylamide gel electrophoresis) was performed with 30 μg of protein per lane using a Mini-PROTEAN System (Bio-Rad, Munich, Germany). For the detection of Akt, gels with a concentration of 12% acrylamide were used, and for the detection of mTOR, gels with an acrylamide concentration of 6%. After electrophoresis, proteins were transferred to PVDF (polyvinylidene difluoride) membranes (Low Fluorescence Membrane Opti Blot, Abcam, Cambridge, GB) using a Mini Trans-Blot Cell (Bio-Rad). Then, the membranes were incubated with antibodies diluted in TBST (Tris-buffered saline with Polysorbate 20: 20 mM Tris, 134 mM NaCl, 0.1% Tween 20 and 2% (w/v) bovine serum albumin; pH 7.6) at the dilutions indicated below.

The primary antibodies used were: mouse anti-pan-AKT [Cell Signaling; #2920] 1:2000 in TBST; rabbit anti-PSer473-AKT [Cell Signaling; #4058] 1:1000 in TBST; mouse-anti-mTOR [Millipore; # 05–1564] 1:1000 in TBST; rabbit anti-PSer2448-mTOR [Cell Signaling; #2971] 1:1000 in TBST. The secondary antibodies employed (red fluorescent IRDye 680RD Goat anti-Mouse and green fluorescent IRDye 800CW Goat anti-Rabbit; both diluted 1:5000 in TBST) were purchased from LI-COR (LI-COR Biosciences, Lincoln, USA). Membranes were scanned using an Odyssey Imaging System (LI-COR, Bad Homburg, Germany), and band intensities were determined by the Image Studio 5 software (LI-COR).

### Statistical analysis

Statistical analysis was carried out using SPSS (IBM, Armonk, USA; Version: 24.0.0.2 64-bit). For multiple comparisons, a one-way ANOVA with the Games-Howell post hoc test was used. A significance level of p<0.05 was considered to be significant. Relative data resulting from two or more experiments or parameters (normalization to reference) with a separate mean and standard deviation are presented using Gaussian error propagation as described before [24]. For the comparison of data obtained from the densitometric analysis of different Western Blot experiments, a least square method was employed in order to calculate means and standard deviations [28].

## Results

### PDK4 expression under the influence of carnosine, rapamycin and Ly-294,002

**Fig 1:**
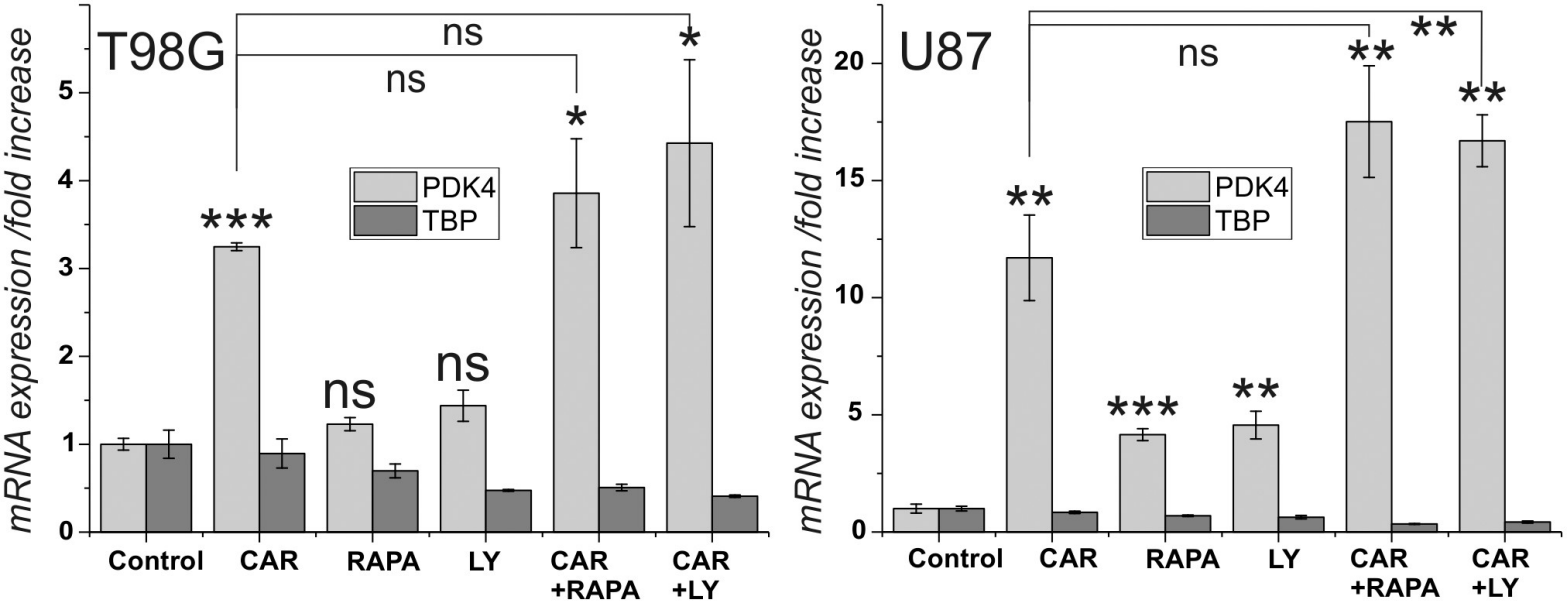
Influence of carnosine, rapamycin and Ly-294,002 on the expression of PDK4 and TBP in cells from the glioblastoma cell lines U87 and T98G. Cells from the lines U87 and T98G were cultivated for 24 hours in the presence of carnosine (Car; 50 mM), rapamycin (Rapa; 25 nM), Ly-294,002 (Ly; 5 μM) or a combination of the compounds. Afterwards expression of PDK4 and TBP was measured and the fold change of expression under the influence of the compounds was determined. Asterisks above the columns indicate a significantly increased expression under the influence of the test compound compared to untreated control cells and the asterisks at the lines connecting columns indicate significant differences between the connected columns: ns: not significant; *: p<0.05; **: p<0.005; ***: p<0.0005.

In a first series of experiments, we asked whether the effect of carnosine on the expression of PDK4 in U87 and T98G glioblastoma cells, that was described by Letzien et al. [24], can also be detected using inhibitors of PI3K/Akt/mTOR signaling. Therefore, cells from the two lines were exposed for 24 hours to carnosine (50 mM), the PI3K inhibitor Ly-294,002 (5 μM), the mTORC1 inhibitor rapamycin (25 nM), and to combinations of the compounds. Then, mRNA was isolated and subjected to RT-qPCR. The result of the experiment is presented in Fig 1. The concentration of rapamycin employed in the experiments (25 nM) has been determined to result in a maximal effect on PDK4 expression in U87 cells, whereas that of Ly-294,002 (5 μM) resulted in an increase of 60% compared to the maximal achievable effect at a concentration of 10 μM in U87 cells (S1 Fig). The rational for using Ly-294,002 at a concentration of 5 μM instead of 10 μM was based on suppliers information that the compound has an IC50 of 0.5 μM/0.57 μM/0.97 μM for PI3Kα/δ/β (Selleckchem, Munich, Germany), but that the compound may also affect targets seemingly unrelated to the PI3K family with an IC50>50 μM (Abcam). Therefore, we preferred to have a concentration as low as possible, but resulting in a reproducible significant effect. As can be seen in Fig 1, carnosine significantly increased the expression of PDK4 in both cell lines, but only in U87 cells a significant increase is seen under the influence of rapamycin and Ly-294,002. Combining Ly-294,002 with carnosine resulted in a higher expression of PDK4, compared to expression with carnosine alone, but only for U87 cells significance was confirmed (p<0.005). Although, the combination of carnosine with rapamycin did also result in a higher expression, compared to that in carnosine alone, this effect was not significant in both cell lines.

### Expression of a PDK4 reporter gene under the influence of carnosine, rapamycin and Ly-294,002

**Fig 2:**
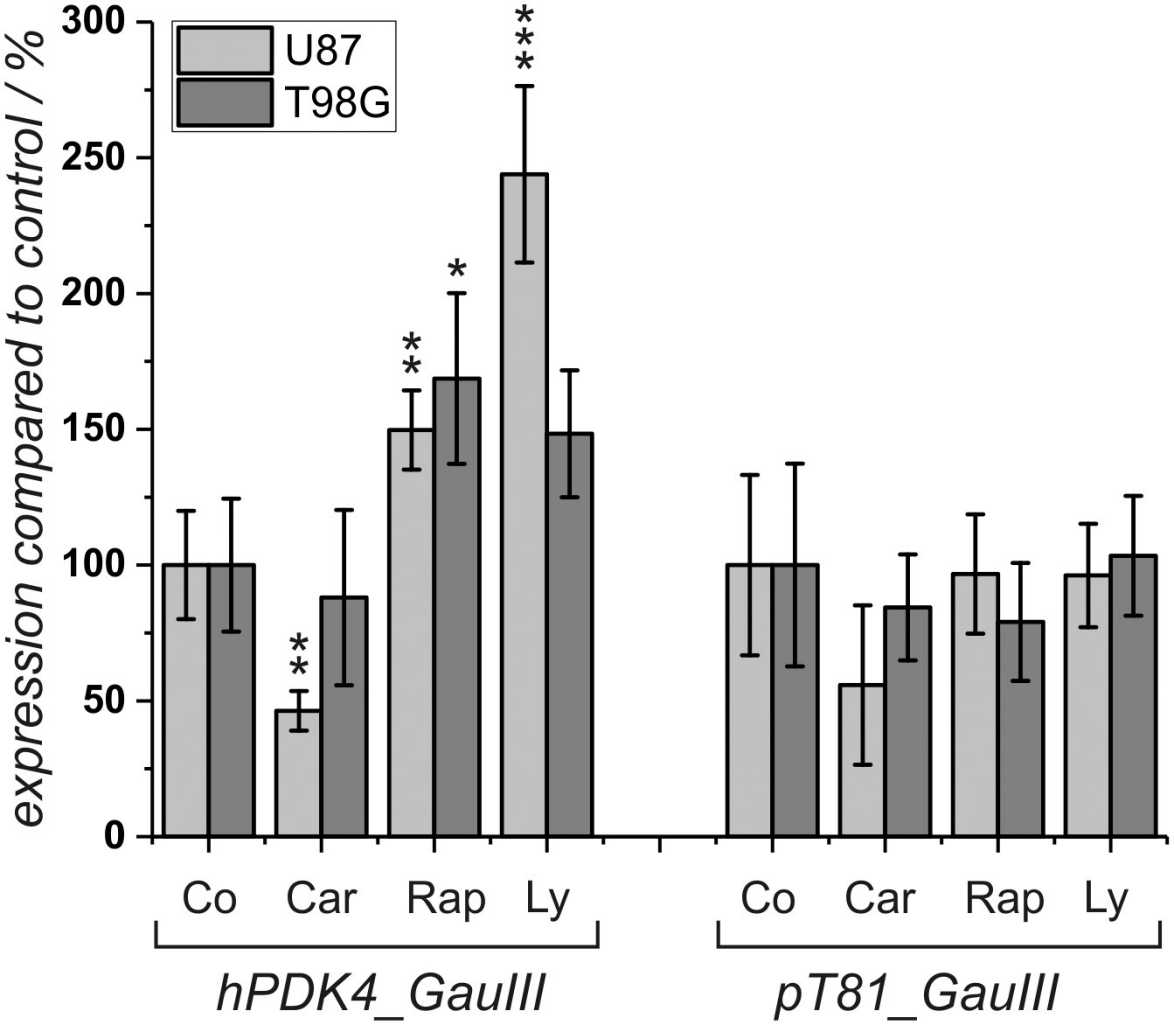
Expression of a reporter gene with human PDK4 5’-region after exposure to different compounds. The reporter gene *hPDK4_GauIII* with the 5’-region from the human PDK4 promoter and the control vector *pT81_GauIII* were transfected into U87 and T98G cells. Cells were exposed to carnosine (Car; 50 mM), rapamycin (Rap; 25 nM), Ly-294,002 (Ly; 5 μM) or to vehicle control (Con) and luciferase activity was determined after 24 hours of incubation (6 independent wells). *: p<0.05; **: p<0.005, and ***: p<0.0005.

**Fig 3:**
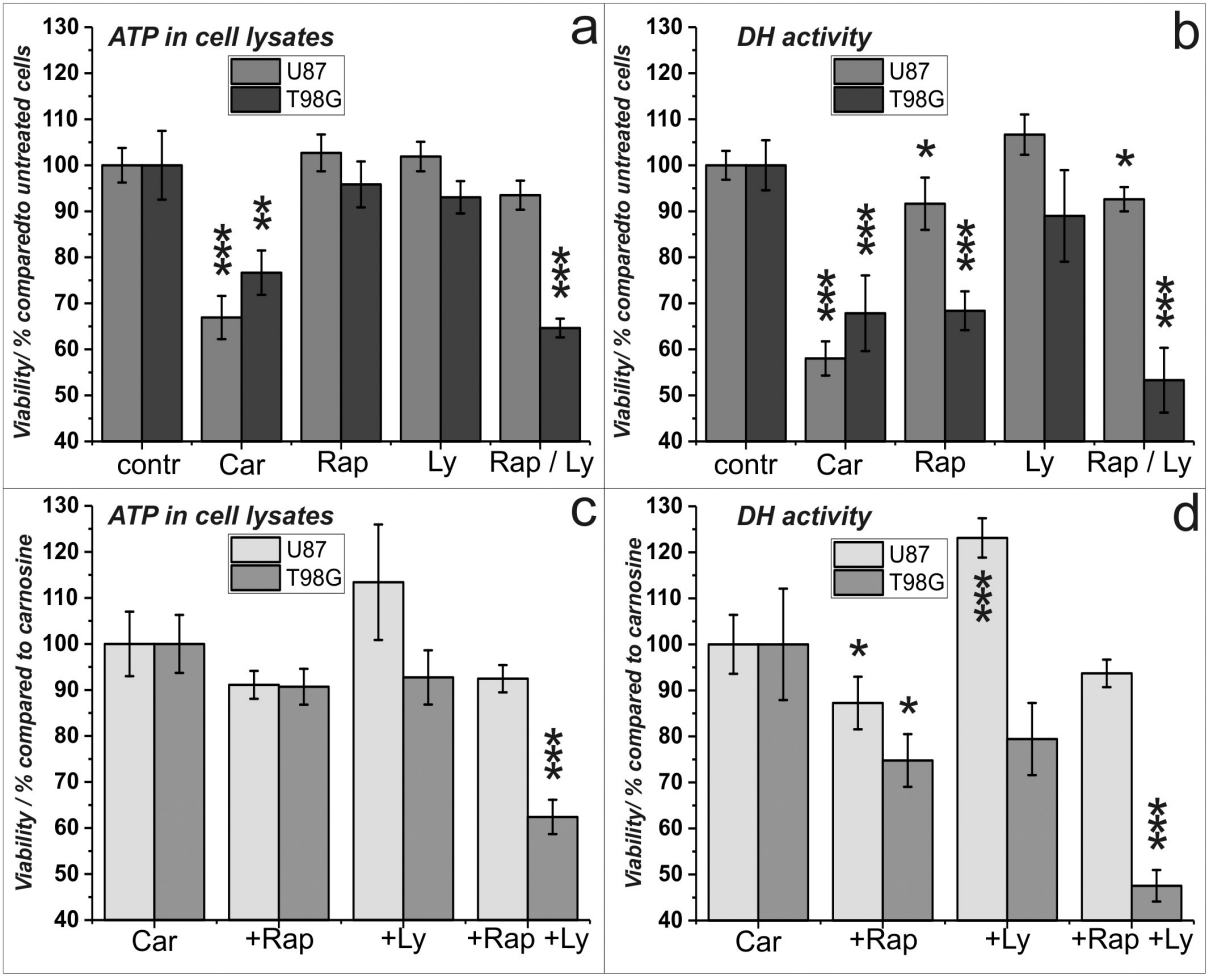
Viability of U87 and T89G cells under the influence of carnosine, rapamycin and Ly-294,002. Cells from the lines U87 and T98G were cultivated for 24 hours in the presence of carnosine (Car; 50 mM), rapamycin (Rap; 25 nM), Ly-294,002 (Ly; 5 μM) or vehicle control (contr). Then, the amount of ATP in cell lysates (left panels) and dehydrogenase activities (right panels) as a measure of cell viability were determined. Upper panels show the influence of each compound on viability compared to untreated cells. In the lower panels the effect of rapamycin and Ly-294,002 on the viability of cells treated in combination with carnosine (cells treated with carnosine only set as 100%) is shown. Mean and standard deviation are presented from 8 independently treated wells for each condition. *: p<0.05; **: p<0.005, and ***: p<0.0005.

As rapamycin and Ly-294,002 were both able to increase expression of PDK4 in the two cell lines investigated (although significance was only confirmed for U87), we wondered whether carnosine exerts its effect on PDK4 expression via PI3/Akt/mTOR signaling. In order to prove this hypothesis, reporter gene assays were performed. The experiments were based on the observation that the effect of PI3K/Akt/mTOR signaling on expression of PDK4 is mediated by elements in the 5’-upstream region of the gene, e.g. by binding sites for transcription factors of the FOXO family [25]. Therefore, we asked whether the effect of carnosine is also mediated by elements located in the promoter of the gene. To answer this question, a reporter gene was constructed that covered a region from the human PDK4 gene (hPDK4) encompassing 3968 bp upstream from the transcriptional start point and 319 bp downstream of it, including the start codon of hPDK4 controlling a luciferase gene with a secretion signal from *Gaussia princeps*. The construct designated “*hPDK4_GauIII*” was transfected into U87 and T98G cells. After 3 hours of exposure to the DNA/transfection complexes, medium was exchanged and the cells were exposed to carnosine (50 mM), rapamycin (25 nM) and Ly-294,002 (5 μM). Twenty-four hours later, luciferase activity was determined from the supernatant. As reference, cells were also transfected with the reporter gene “*pT81_GauIII*” used for the construction of “*hPDK4_GauIII*” which only contained a minimal promoter from the thymidine kinase of *Herpes simplex*. The result of a corresponding experiment is presented in Fig 2. The experiment demonstrates a significant response of the PDK4 reporter gene to the presence of rapamycin in both cell lines. In the presence of Ly-294,002, we also observed increased expression in both cell lines but statistical significance could only be confirmed for U87 cells. Regarding expression of the corresponding control vector, no statistically significant influence of carnosine, rapamycin, and Ly-294,002 was obtained. In the presence of carnosine, the reporter gene and the control plasmid exhibited a comparable reduced expression in U87 cells (statistically significant only for the reporter gene) and no response in cells from the line T98G. Note: The negative response in the presence of carnosine, which becomes significant in U87 cells transfected with the reporter gene, is a result of reduced viability under the influence of the dipeptide (compare Fig 3). Therefore, we conclude that carnosine’s effect on expression of the endogenous PDK4 gene is independent from an interaction of transcription factors within the tested cis-elements and also different from the effects exhibited by rapamycin and Ly-294,002.

### Cell viability under the influence of carnosine, rapamycin and Ly-294,002

As the reporter gene assay performed indicated a strong influence of carnosine on viability of cells from the line U87, which was not observed in the presence of rapamycin and Ly-294,002, we decided to study the effect of the three compounds on viability in more detail. Therefore, U87 and T98G cells were cultivated in 96-well plates and exposed to carnosine (50 mM), rapamycin (25 nM) and Ly-294,002 (5 μM). Twenty-four hours later, cell viability was determined measuring ATP in cell lysates using the CellTiter-Glo assay and dehydrogenase activity using the CellTiterBlue assay. The result of an experiment in which 5000 cells per well were used is presented in Fig 3. As can be seen, carnosine had a significant effect on viability of both cell lines as determined by ATP production (Fig 3a) and dehydrogenase activity (Fig 3b). The determination of ATP in cell lysates did not show an influence of rapamycin on viability, but the determination of dehydrogenase activity clearly indicated that rapamycin affects dehydrogenase activity as a measure of viability, which is more prominent in cells from the line T98G than in cells from the line U87. Analyzing effects by combination of carnosine with the other compounds (Fig 3c/3d), a significant reduction of viability compared to cells treated with carnosine alone was detected by combination with rapamycin in both cell lines but only regarding dehydrogenase activity. Interestingly, dehydrogenase activity was increased in U87 cells treated with Ly-294,002 and carnosine compared to carnosine alone, and we identified a reduction of dehydrogenase activity in T98G cells combining Ly-294,002 with carnosine relative to cells treated with carnosine alone, which was not significant.

### Phosphorylation of Akt and mTOR under the influence of carnosine, rapamycin and Ly-294,002

**Fig 4:**
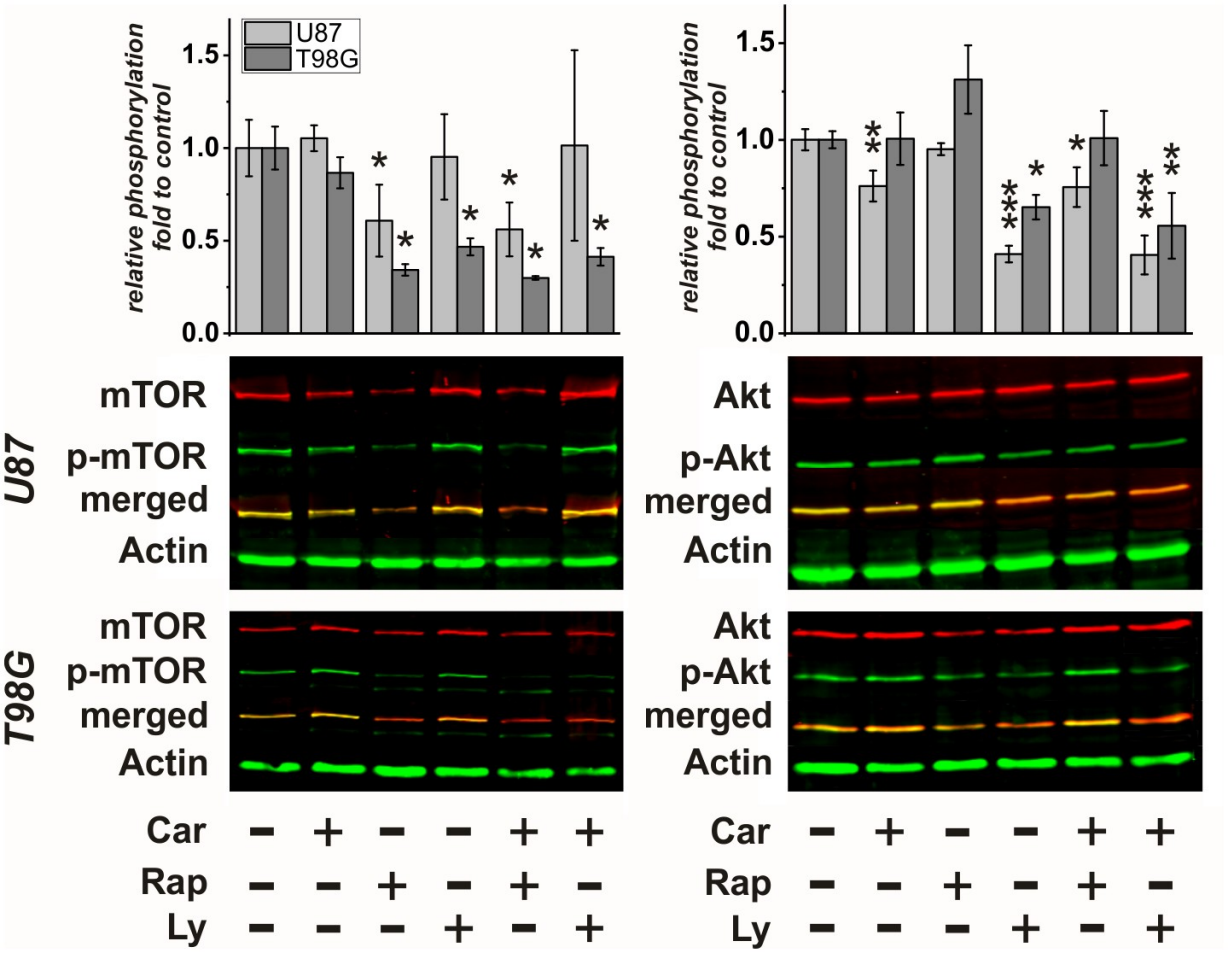
Phosphorylation of mTOR and Akt under the influence of carnosine, rapamycin and Ly-294,002. Cells from the lines U87 and T98G were incubated in the presence of carnosine (Car; 50 mM), rapamycin (Rap; 25 nM), Ly-294,002 (Ly; 5 μM) or vehicle control or a combination of the compounds. Proteins were isolated, subjected to SDS-PAGE and Western Blotting was performed using antibodies for mTOR and mTOR phosphorylation at serine 2448 (mTOR and p-mTOR; left) and antibodies against Akt and Akt phosphorylation at serine 473 (Akt and p-Akt right). In the lower part, representative blots are presented in which the total amount of each signaling molecule is detected by red fluorescence and its corresponding phosphorylated form by green fluorescence. Below the combined bands are shown (merged) in yellow. Actin served as loading control (green fluorescence). A densitometry analysis from six (U87) and three (T98G) independent experiments was performed with each set of the two primary antibodies. Data is presented as “relative phosphorylation”, indicating the ratio of the signal obtained with the phospho-specific antibody compared to the signal with the antibody direct against total protein of the factor under the influence of a compound, compared to this ratio in untreated control cells (set as 1). Statistical significance was calculated by one-way ANOVA with the Games-Howell post hoc test: *: p<0.05; **: p<0.005, and ***: p<0.0005.

The experiments presented in the preceding paragraphs indicated that carnosine’s effect on PDK4 expression is different from that of rapamycin and Ly-294,002. To finally analyze whether PI3K/Akt/mTOR pathway signaling in U87 and T98G cells is affected at the level of Akt and mTOR phosphorylation, when the cells are exposed to carnosine and the inhibitors, we performed Western Blot experiments. Therefore, U87 and T98G cells were cultivated for 24 hours in the presence of carnosine (50 mM), rapamycin (25 nM) and Ly-294,002 (5 μM), and combinations of the compounds. Then, proteins were extracted, subjected to SDS-PAGE and Western Blotting was performed with antibodies directed against mTOR and Akt and their phosphorylated forms. In Fig 4 representative Blots and a densitometric analysis from six (U87), and three (T98G) independent experiments with each pair of antibodies are presented. Whereas rapamycin was able to reduce mTOR phosphorylation in both cell lines, Ly-294,002 did affect phosphorylation of mTOR only in T98G cells but not in U87 cells. Most importantly, carnosine had no effect on phosphorylation of mTOR. Analyzing the phosphorylation of Akt we did not detect an effect of rapamycin on its phosphorylation, but a significant one by Ly-294,002 in both cell lines. Interestingly, carnosine significantly reduced phosphorylation of Akt in cells from the line U87 but not in T98G.

## Discussion

Here, we investigated, whether carnosine has an influence on the PI3K/Akt/mTOR pathway. Ample laboratory studies suggest that this pathway is vital to the growth and survival of cancer cells, including glioblastoma with hyperactivated PI3K/Akt/mTOR [7]. In our experiments, the PI3Kα/δ/β inhibitor Ly294,002 significantly inhibited phosphorylation of Akt in T98G and U87 cells, but an effect on phosphorylation of mTOR after 24 hours of treatment was only detected in T98G and not in U87 cells. More importantly, we found no indication of reduced viability after treatment with Ly294,002 in both cell lines. This appears to be in contrast to results obtained by others [29]. These authors observed enhanced survival of nude mice with intracranially implanted U87 cells after oral administration of the pan-class I PI3K inhibitor NVP-BKM120 (buparsilib; 5 times a week for a total of 20 treatments). In addition, they also detected enhanced apoptosis, as detected by Annexin V staining, in cultured U87 cells after 72 hour treatment with NVP-BKM120 [29]. We assume that it may be that the 24-hour incubation period employed in our experiments is too short to see effects of PI3K inhibition on tumor cell viability. This assumption is supported by data obtained with the glioblastoma cell lines LN229 and U251, which did significantly respond to 10 μM Ly294,002 only when they were incubated in its presence for more than one day [30]. It also should be noted that other authors point out that in the range of 4 to 8 μM Ly294,002 does not discernibly affect cell cycle progression, at least in U251 MG glioblastoma cells [31]. Carnosine, which also reduced phosphorylation of Akt in U87 cells, although not as strong as Ly294,002, had a strong effect on viability already after 24-hour exposure. We currently do not know why phosphorylation of Akt under the influence of carnosine is not observed in T98G cells. However, it has to be taken into account, that the status of phosphorylation is also dependent on the activity of phosphatases and other mechanisms such as subcellular localization (for a recent review see [32]).

Rapamycin (also known as Sirolimus) did reduce phosphorylation of mTOR in both cell lines, as it had to be expected, but it had no effect on viability as determined by ATP in cell lysates. Measuring dehydrogenase activity, a minor effect of rapamycin on viability was detected in U87 cells. In T98G cells the effect of rapamycin was comparable to the effect of carnosine, when measuring dehydrogenase activity. From these data, we conclude that the anti-neoplastic effect of carnosine is certainly not mediated via an effect on PI3K, and also unlikely to be mediated via mTOR. However, as demonstrated by the data presented in Fig 3, a small combinatorial effect of carnosine and rapamycin can be observed in both cell lines with regard to dehydrogenase activity, and not with regard to the amount of ATP in cell lysates.

The influence of Ly294,002 and rapamycin on PDK4 expression is known to be mediated by 5’-elements in the PDK4 gene, which we confirmed by our reporter gene assay. As carnosine did not increase the expression of the reporter gene, this observation supports the assumption resulting from the viability assays, that carnosine must mediate its physiological effects without influencing PI3K/Akt/mTOR in the glioblastoma cells used. At this point it may be hypothesized that elements which were not present in our reporter gene, such as intronic or 3’-elements, could be responsible for the induction of the PDK4 gene under the influence of carnosine. In fact, we just recently demonstrated that the effect of carnosine on PDK4 expression is linked to epigenetic mechanisms [33]. At this point it is also interesting to note, that a recent report, comparing PDK4 mRNA expression under the influence of carnosine and L-histidine in 21 primary glioblastoma cultures and 10 glioblastoma cell lines, revealed that no correlation exists between increased mRNA expression and the compounds influence on viability [34].

As already outlined in the introduction, there is evidence, that carnosine’s effect on tumor cell viability is accompanied by effects on signal transduction and on tumor cell metabolism. However, the question remains, whether the effects on signal transduction detected in other tumor models, as outlined in the introduction, are resulting from a direct effect on a single transduction molecule or on a receptor. In fact, we recently demonstrated that increased expression of PDK4 is accompanied by epigenetic regulation [33], and the possibility cannot be ruled out that the primary target of carnosine is an unknown Histone Deacetylase. In that case, the observed changes in signal transduction may be a secondary response. As carnosine is able to react non-enzymatically with aldehydes [35], one could also imagine that its primary target is a metabolite, such as an aldehyde of the glycolytic pathway, which might explain its effect on ATP production from glucose [17]. Any influence on metabolism will in turn also feed-back on signal transduction [36].

Although, carnosine’s primary targets have still not been revealed, we consider it worth to further investigate them, as understanding carnosine’s mode of action could pave the way to the discovery of novel therapeutic interventions. In this context, it should be emphasized, that the present investigation demonstrates, that tumors, which do not or less respond to drugs targeting pathways, such as the PI3K/Akt/mTOR pathway, are still sensitive to carnosine. This could offer the opportunity for new strategies for the treatment of tumors in general and glioblastoma in particular, that are resistant to currently employed drugs. However, these interventions may include the design of novel drugs, targeting the primary targets of carnosine, as we are aware, that the concentrations employed in the in vitro experiments in the present study may not be reached in a patient by oral administration of the dipeptide. On the other hand, dose response studies demonstrated that lower concentrations are also effective [14,24], especially after prolonged incubation [16]. In addition, the great advantage of carnosine is, that it is a naturally occurring compound, which has already been used for the treatment of other diseases, without having side-effects [37–39]. In addition, we just recently demonstrated that the dipeptide does not decrease the viability of fibroblasts [16], and does even have neuroprotective effects [40].

## Supporting information

S1 Fig Relative PDK4 expression after exposure to different concentrations of rapamycin and Ly-294,002. Cells from the line U87 were cultivated at a density of 10^6^ cells per culture plate and exposed for 24 hours to different concentrations of rapamycin and Ly-294,002. The fold of enhancement of PDK4 mRNA expression was calculated from the relative expression of PDK4 (compared to the expression of mRNA encoding TBP) and compared to the corresponding control cells treated with vehicle.
(TIF)

## References

[1] Ostrom QT, Gittleman H, Truitt G, Boscia A, Kruchko C, Barnholtz-Sloan JS. CBTRUS Statistical Report: Primary Brain and Other Central Nervous System Tumors Diagnosed in the United States in 2011–2015. Neuro-Oncology. 2018; 20: iv1–iv86. 10.1093/neuonc/noy131 30445539 PMC6129949

[2] Stupp R, Mason WP, van den Bent M. J., Weller M, Fisher B, Taphoorn MJB, et al Radiotherapy plus concomitant and adjuvant temozolomide for glioblastoma. New England Journal of Medicine. 2005; 352: 987–996. 10.1056/NEJMoa043330 15758009

[3] Wen PY, Kesari S. Malignant gliomas in adults. N Engl J Med. 2008; 359: 492–507. 10.1056/NEJMra0708126 18669428

[4] Bush NAO, Chang SM, Berger MS. Current and future strategies for treatment of glioma. Neurosurg Rev. 2017; 40: 1–14. 10.1007/s10143-016-0709-8 27085859

[5] Alifieris C, Trafalis DT. Glioblastoma multiforme. Pathogenesis and treatment. Pharmacol Ther. 2015; 152: 63–82. 10.1016/j.pharmthera.2015.05.005 25944528

[6] Li X, Wu C, Chen N, Gu H, Yen A, Cao L, et al PI3K/Akt/mTOR signaling pathway and targeted therapy for glioblastoma. Oncotarget. 2016 10.18632/oncotarget.7961 26967052 PMC5078108

[7] Zhao H-F, Wang J, Shao W, Wu C-P, Chen Z-P, To S-ST, et al Recent advances in the use of PI3K inhibitors for glioblastoma multiforme. Current preclinical and clinical development. Mol Cancer. 2017; 16: 100 10.1186/s12943-017-0670-3 28592260 PMC5463420

[8] Gulewitsch W, Amiradzibi S. Ueber das Carnosin, eine neue organische Base des Fleischextraktes. Ber Dtsch Chem Ges. 1900; 33: 1902–1903.

[9] Mannion AF, Jakeman PM, Dunnett M, Harris RC, Willan PL. Carnosine and anserine concentrations in the quadriceps femoris muscle of healthy humans. Eur J Appl Physiol Occup Physiol. 1992; 64: 47–50. 1735411 10.1007/BF00376439

[10] Boldyrev AA, Aldini G, Derave W. Physiology and pathophysiology of carnosine. Physiol Rev. 2013; 93: 1803–1845. 10.1152/physrev.00039.2012 24137022

[11] Horii Y, Shen J, Fujisaki Y, Yoshida K, Nagai K. Effects of l-carnosine on splenic sympathetic nerve activity and tumor proliferation. Neurosci Lett. 2012; 510: 1–5. 10.1016/j.neulet.2011.12.058 22240100

[12] Shen Y, Yang J, Li J, Shi X, Ouyang L, Tian Y, et al Carnosine inhibits the proliferation of human gastric cancer SGC-7901 cells through both of the mitochondrial respiration and glycolysis pathways. PLoS ONE. 2014; 9: e104632 10.1371/journal.pone.0104632 25115854 PMC4130552

[13] Ditte Z, Ditte P, Labudova M, Simko V, Iuliano F, Zatovicova M, et al Carnosine inhibits carbonic anhydrase IX-mediated extracellular acidosis and suppresses growth of HeLa tumor xenografts. BMC Cancer. 2014; 14: 358 10.1186/1471-2407-14-358 24886661 PMC4061103

[14] Renner C, Seyffarth A, de Arriba S, Meixensberger J, Gebhardt R, Gaunitz F. Carnosine Inhibits Growth of Cells Isolated from Human Glioblastoma Multiforme. Int J Pept Res Ther. 2008; 14: 127–135. 10.1007/s10989-007-9121-0

[15] Renner C, Zemitzsch N, Fuchs B, Geiger KD, Hermes M, Hengstler J, et al Carnosine retards tumor growth in vivo in an NIH3T3-HER2/neu mouse model. Mol Cancer. 2010; 9: 2 10.1186/1476-4598-9-2 20053283 PMC2818694

[16] Oppermann H, Dietterle J, Purcz K, Morawski M, Eisenlöffel C, Müller W, et al Carnosine selectively inhibits migration of IDH-wildtype glioblastoma cells in a co-culture model with fibroblasts. Cancer Cell Int. 2018; 18: 111 10.1186/s12935-018-0611-2 30123089 PMC6090706

[17] Oppermann H, Schnabel L, Meixensberger J, Gaunitz F. Pyruvate attenuates the anti-neoplastic effect of carnosine independently from oxidative phosphorylation. Oncotarget. 2016; 7: 85848–85860. 10.18632/oncotarget.13039 27811375 PMC5349879

[18] Iovine B, Oliviero G, Garofalo M, Orefice M, Nocella F, Borbone N, et al The Anti-Proliferative Effect of L-Carnosine Correlates with a Decreased Expression of Hypoxia Inducible Factor 1 alpha in Human Colon Cancer Cells. PLoS One. 2014; 9: e96755 10.1371/journal.pone.0096755 24804733 PMC4013086

[19] Wang J-P, Yang Z-T, Liu C, He Y-H, Zhao S-S. L-carnosine inhibits neuronal cell apoptosis through signal transducer and activator of transcription 3 signaling pathway after acute focal cerebral ischemia. Brain Res. 2013; 1507: 125–133. 10.1016/j.brainres.2013.02.032 23454231

[20] Kulebyakin K, Karpova L, Lakonsteva E, Krasavin M, Boldyrev A. Carnosine protects neurons against oxidative stress and modulates the time profile of MAPK cascade signaling. Amino Acids. 2012; 43: 91–96. 10.1007/s00726-011-1135-4 22101981

[21] Iovine B, Iannella ML, Nocella F, Pricolo MR, Baldi MR, Bevilacqua MA. Carnosine inhibits KRas-mediated HCT-116 proliferation by affecting ATP and ROS production. Cancer Lett. 2011; 315: 122–128. 10.1016/j.canlet.2011.07.021 22137144

[22] Zhang Z, Miao L, Wu X, Liu G, Peng Y, Xin X, et al Carnosine Inhibits the Proliferation of Human Gastric Carcinoma Cells by Retarding Akt/mTOR/p70S6K Signaling. J Cancer. 2014; 5: 382–389. 10.7150/jca.8024 24799956 PMC4007526

[23] Hipkiss AR. Energy metabolism, proteotoxic stress and age-related dysfunction–Protection by carnosine. Mol Aspects Med. 2011; 32: 267–278. 10.1016/j.mam.2011.10.004 22020113

[24] Letzien U, Oppermann H, Meixensberger J, Gaunitz F. The antineoplastic effect of carnosine is accompanied by induction of PDK4 and can be mimicked by L-histidine. Amino Acids. 2014 10.1007/s00726-014-1664-8 24398899

[25] Kwon H-S, Huang B, Unterman TG, Harris RA. Protein kinase B-alpha inhibits human pyruvate dehydrogenase kinase-4 gene induction by dexamethasone through inactivation of FOXO transcription factors. Diabetes. 2004; 53: 899–910. 10.2337/diabetes.53.4.899 15047604

[26] Braun S, Oppermann H, Mueller A, Renner C, Hovhannisyan A, Baran-Schmidt R, et al Hedgehog signaling in glioblastoma multiforme. cbt. 2012; 13: 487–495. 10.4161/cbt.19591 22406999

[27] Gaunitz F, Heise K. HTS compatible assay for antioxidative agents using primary cultured hepatocytes. Assay.Drug Dev.Technol. 2003; 1: 469–477. 10.1089/154065803322163786 15090184

[28] Degasperi A, Birtwistle MR, Volinsky N, Rauch J, Kolch W, Kholodenko BN. Evaluating strategies to normalise biological replicates of Western blot data. PLoS ONE. 2014; 9: e87293 10.1371/journal.pone.0087293 24475266 PMC3903630

[29] Koul D, Fu J, Shen R, LaFortune TA, Wang S, Tiao N, et al Antitumor activity of NVP-BKM120—a selective pan class I PI3 kinase inhibitor showed differential forms of cell death based on p53 status of glioma cells. Clinical Cancer Research. 2012; 18: 184–195. 10.1158/1078-0432.CCR-11-1558 22065080 PMC3785365

[30] Nan Y, Guo L, Song Y, Le Wang, Yu K, Huang Q, et al Combinatorial therapy with adenoviral-mediated PTEN and a PI3K inhibitor suppresses malignant glioma cell growth in vitro and in vivo by regulating the PI3K/AKT signaling pathway. J Cancer Res Clin Oncol. 2017; 143: 1477–1487. 10.1007/s00432-017-2415-5 28401302 PMC11819009

[31] Nakamura JL, Karlsson A, Arvold ND, Gottschalk AR, Pieper RO, Stokoe D, et al PKB/Akt mediates radiosensitization by the signaling inhibitor LY294002 in human malignant gliomas. J Neurooncol. 2005; 71: 215–222. 10.1007/s11060-004-1718-y 15735908

[32] Yudushkin I. Getting the Akt Together: Guiding Intracellular Akt Activity by PI3K. Biomolecules. 2019; 9 10.3390/biom9020067 30781447 PMC6406913

[33] Oppermann H, Alvanos A, Seidel C, Meixensberger J, Gaunitz F. Carnosine influences transcription via epigenetic regulation as demonstrated by enhanced histone acetylation of the pyruvate dehydrogenase kinase 4 promoter in glioblastoma cells. Amino Acids. 2018 10.1007/s00726-018-2619-2 30030619

[34] Oppermann H, Purcz K, Birkemeyer C, Baran-Schmidt R, Meixensberger J, Gaunitz F. Carnosine’s inhibitory effect on glioblastoma cell growth is independent of its cleavage. Amino Acids. 2019: 761–772. 10.1007/s00726-019-02713-6 30863889

[35] da Silva Bispo V, Di Mascio P, Medeiros M. Quantification of Carnosine-Aldehyde Adducts in Human Urine. Free Radic Biol Med. 2014; 75 Suppl 1: S27 10.1016/j.freeradbiomed.2014.10.751 26461323

[36] Metallo CM, Vander Heiden MG. Metabolism strikes back: metabolic flux regulates cell signaling. Genes Dev. 2010; 24: 2717–2722. 10.1101/gad.2010510 21159812 PMC3003187

[37] Chez MG, Buchanan CP, Aimonovitch MC, Becker M, Schaefer K, Black C, et al Double-blind, placebo-controlled study of L-carnosine supplementation in children with autistic spectrum disorders. J Child Neurol. 2002; 17: 833–837. 10.1177/08830738020170111501 12585724

[38] Boldyrev A, Fedorova T, Stepanova M, Dobrotvorskaya I, Kozlova E, Boldanova N, et al Carnosine [corrected] Increases Efficiency of DOPA Therapy of Parkinson’s Disease: A Pilot Study. Rejuvenation Res. 2008; 11: 821–827. 10.1089/rej.2008.0716 18729814

[39] Baraniuk JN, El-Amin S, Corey R, Rayhan R, Timbol C. Carnosine Treatment for Gulf War Illness: A Randomized Controlled Trial. GJHS. 2013; 5 10.5539/gjhs.v5n3p69 23618477 PMC4209301

[40] Devyatov AA, Fedorova TN, Stvolinsky SL, Ryzhkov IN, Riger NA, Tutelyan VA. Issledovanie neĭroprotektornykh mekhanizmov deĭstviia karnozina pri éksperimental’noĭ fokal’noĭ ishemii/reperfuzii. Biomed Khim. 2018; 64: 344–348. 30135281 10.18097/PBMC20186404344

