# Supplementary figures and images for: Carnosine inhibits glioblastoma growth independent from PI3K/Akt/mTOR signaling
Source: PLoS One. 2019 Jun 27;14(6):e0218972. doi: 10.1371/journal.pone.0218972 (PMC6597087; doi:10.1371/journal.pone.0218972)

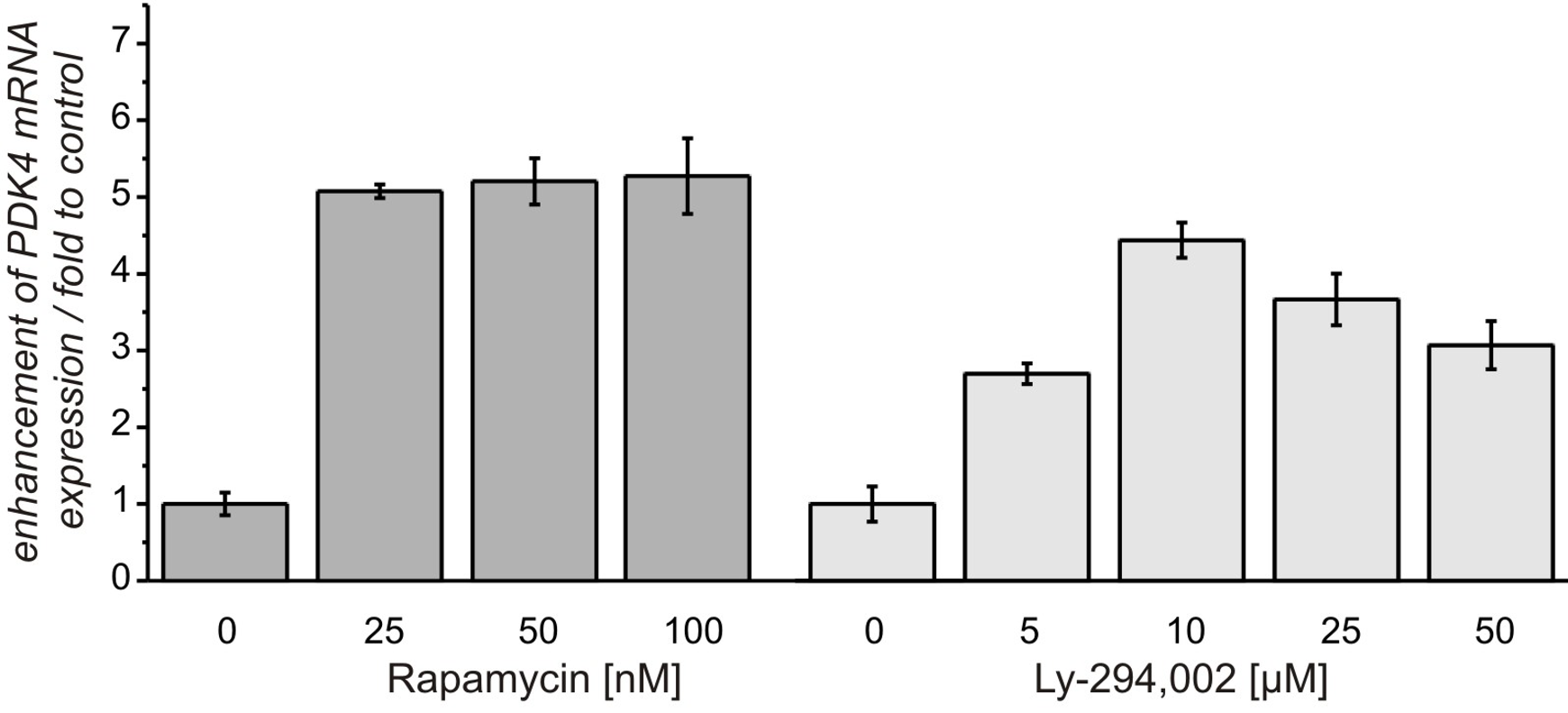

Supplement: S1 Fig — Cells from the line U87 were cultivated at a density of 106 cells per culture plate and exposed for 24 hours to different concentrations of rapamycin and Ly-294,002. The fold of enhancement of PDK4 mRNA expression was calculated from the relative expression of PDK4 (compared to the expression of mRNA encoding TBP) and compared to the corresponding control cells treated with vehicle. (TIF) [file pone.0218972.s001.tif]
